# Prevalence of hookah smoking among Iranian pupils and university students: An updated systematic review and meta‐analysis

**DOI:** 10.1111/crj.13511

**Published:** 2022-06-03

**Authors:** Hamid Zaheri, Yosra Raziani, Nesa Khademi, Yousef Moradi, Hossein Shahriari, Reza Ghanei‐Gheshlagh

**Affiliations:** ^1^ Student Research Committee Kurdistan University of Medical Sciences Sanandaj Iran; ^2^ Nursing Department Komar University of Science and Technology Sulaymaniyah Iraq; ^3^ Student Research Committee Lorestan University of Medical Sciences Khorramabad Iran; ^4^ Department of Epidemiology and Biostatistics, Faculty of Medicine Kurdistan University of Medical Sciences Sanandaj Iran; ^5^ Faculty of Para Medicine Shahid Beheshti University of Medical Sciences Tehran Iran; ^6^ Social Determinants of Health Research Center, Research Institute for Health Development Kurdistan University of Medical Sciences Sanandaj Iran

**Keywords:** hookah, Iran, prevalence, pupil, student

## Abstract

**Objective:**

Today, smoking is considered a pressing global health issue. The present study aimed to estimate the total prevalence of hookah smoking among pupils and university students in Iran.

**Materials and Methods:**

This systematic review and meta‐analysis were conducted via searching in databases such as Scientific Information Database (SID), MagIran, Scopus, PubMed and Web of Sciences from inception to October 2021. We targeted observational studies evaluating the prevalence or frequency of hookah smoking among Iranian pupils and university students. Data analysis was performed using a random‐effects model, and the heterogeneity of the articles was assessed using Cochran's *Q* test and the *I* ^2^ statistic.

**Results:**

In total, 124 studies conducted on 155 115 subjects were reviewed. The lifetime prevalence of hookah smoking among high school students and university students was estimated at 34.4% and 32.3%, respectively. In addition, the frequency of hookah smoking within the past month/week (point prevalence) was estimated at 21.5% and 16.6% in university students and pupils, respectively. The frequency of hookah smoking within the past year (period prevalence) was also reported to be 22.5% and 20.8% in these groups, respectively. No significant correlation was observed between the prevalence of hookah smoking, sample size, year of publication and the mean age of the participants. Region 5 had the highest lifetime prevalence (41.7%) and period prevalence (27.1%). However, Region 1 had the highest point prevalence of hookah smoking (27.2%).

**Conclusions:**

According to the results, hookah smoking is highly prevalent among Iranian pupils and university students. Therefore, proper educational interventions are required in the form of workshops and curricula to raise awareness regarding the hazardous effects of this unhealthy habit on the young generation.

## Graphical abstract

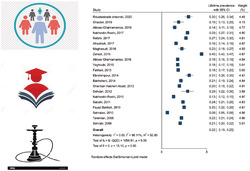

## 1 INTRODUCTION

Smoking is a leading cause of premature and preventable deaths in the world. Each year, more than eight million die due to the diseases caused by tobacco consumption across the globe, which costs the global economy 1.4 billion dollars.^1^ Hookah is a traditional way of smoking, which has gained popularity among pupils and students, currently becoming an epidemic.^2^ The risks of hookah smoking are often undermined due to various misconceptions, such as the use of flavouring agents in tobacco products and the false public opinion that hookah is healthy because the hazardous agents in the tobacco are filtered through the water inside the tank.^3^ Nonetheless, hookah is similar to cigarettes in terms of causing diseases such as cancer, respiratory and gastrointestinal disorders, chronic pulmonary diseases and cardiovascular diseases.^4^, ^5^ Furthermore, the persistent use of hookah could lead to nicotine dependency.^6^, ^7^

Statistics suggest that the prevalence of hookah smoking is 4% to34% worldwide.^8^ In a comprehensive study conducted on 105 000 university students in the United States, the prevalence of hookah smoking was reported to be 8.4% within the past month.^9^ In another research in the United States, 19 000 students were evaluated, and the recent prevalence rate (past 30 days) of hookah smoking was estimated at 3.4% in high school students.^10^ Different results have been reported in the studies conducted on Iranian pupils and university students. Because smoking habits often initiate in adolescence,^11^ the first step to the prevention of this issue is to identify the status of hookah smoking in the Iranian community so that proper interventions and managerial strategies could be developed in this regard. The present study aimed to estimate the prevalence of hookah smoking among Iranian pupils and university students.

## 2 MATERIALS AND METHODS

This systematic review and meta‐analysis aimed to determine the lifetime prevalence rate of hookah smoking in Iranian pupils and university students based on the Preferred Reporting Items for Systematic Reviews and Meta‐Analyses (PRISMA) guidelines. The protocol of this study has been sent to PROSPERO and is under review.

### 2.1 Search strategy

A literature search was conducted in databases such as the Scientific Information Database (SID), MagIran, Scopus, PubMed and Web of Science from inception to October 2021. The keywords used in the database search included (“Smoking Water Pipes” OR “Smoking Water Pipe” OR “Hooka*” OR “Shisha*” OR “Sheesha*” OR “Smoking Waterpipe*” OR “Narghile*”) AND (“Prevalence” OR “Prevalence*” R “Period Prevalence*” OR “Point Prevalence*”) AND (“students” OR “student*” OR “School Enrollment*”) AND (“Iran” OR “iran*”).

Because the national databases were not sensitive to Boolean operators, the database search was performed in a single‐word manner. To retrieve more eligible papers and have access to ‘grey literature’, the references lists of the identified articles were also manually searched. The literature search was conducted during 20 September–20 October 2021. Selected articles were analysed in the EndNote software version 8. Following that, the papers were screened based on title, abstract and full text. The screening stages were carried out by two authors independently. In case of disagreement, a third author would resolve the matter. Finally, all the authors attended a meeting in which the final articles were put to discussion to be selected based on the inclusion and exclusion criteria.

### 2.2 Inclusion and exclusion criteria

The inclusion criteria were observational studies published in English and Persian regarding the prevalence of hookah smoking among Iranian pupils and university students. Interventional studies, reviews, letters to the editor, qualitative/analytical studies, care reports and cohorts without essential data were excluded from the review.

### 2.3 Data extraction

Two authors extracted data on the authors' names, year of publication, sample size, mean age of the participants, study area (city), target population (pupils/university students) and the prevalence of hookah smoking.

### 2.4 Qualitative assessment of the selected articles

Two authors qualitatively assessed the retrieved articles based on the Newcastle–Ottawa quality assessment scale (NOS). The NOS checklist is used to qualitatively assess observational studies, especially cross‐sectional studies. The NOS evaluates articles based on six items in three dimensions of sample collection method, comparison and analysis of study groups, and the outcome measurement and analysis. If these criteria are met, each item is scored one, and the maximum achievable score for each article is nine. In the current review, discrepancies in the allocated score to the selected articles were resolved by the third author through discussion to reach a consensus.^12^

### 2.5 Statistical analysis

To perform the meta‐analysis, the prevalence rates extracted from the selected studies were calculated based on standard error. The random‐effects model by DerSimonian–Liard was also employed to estimate the cumulative prevalence of hookah smoking (95% confidence interval [CI]) using the Metaprop and Metan commands in the Stata software version 16. To assess the homogeneity and variance of the selected studies for the meta‐analysis, we used Cochran's *Q* test and the *I* ^2^ statistic. Moreover, publication bias was evaluated using the funnel plot and Egger's test. Meta‐regression analysis was also utilized to determine the correlations of age, sample size and year of publication with the prevalence of hookah smoking. In all the statistical tests, we considered *α* = 0.05.

## 3 RESULTS

**FIGURE 1:**
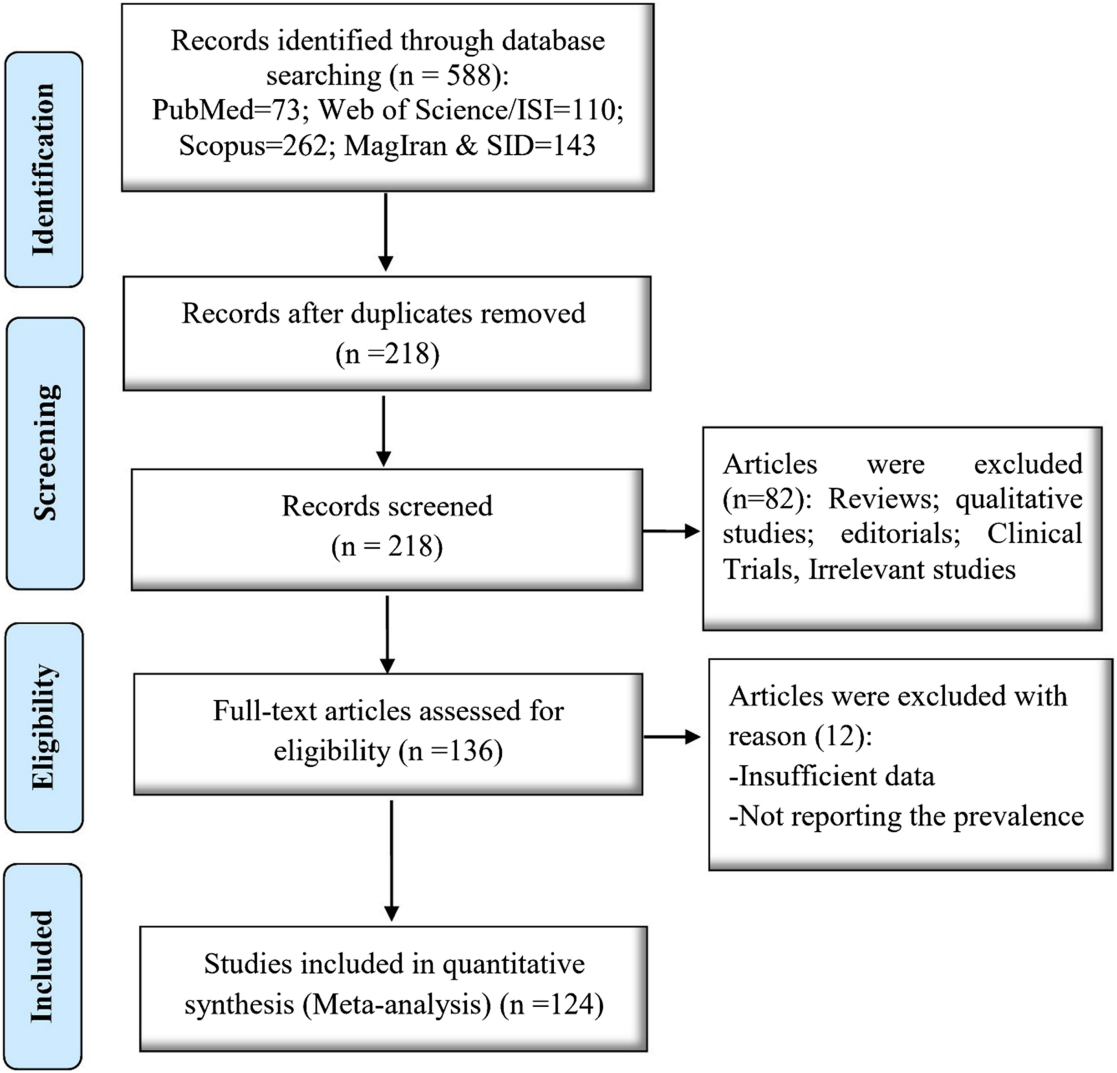
Article selection process

An initial search yielded 588 articles. After removing duplicates, the titles and abstracts of the remaining 218 articles were evaluated. Based on the inclusion criteria, irrelevant studies were excluded and the full text of the remaining articles was studied (Figure 1).

In total, 124 eligible studies were selected for the current review, which had been conducted on 155 115 subjects (mean per study: 1251). The studies were performed on high school and university students. In addition, 44.5% of the studies had a larger sample size than 1000. Most of the studies (28.2%) were conducted in Region 1 of the country. The oldest and most recent studies were published in 2001 (*n* = 1) and 2021 (*n* = 5), respectively (Table 1).

**TABLE 1:** The characteristics of selected papers

First author	Year	Sample size	Place	Age	P‐last year (period prevalence)	P‐last month/week (point prevalence)	P‐experience (lifetime prevalence)
Bashirian^13^	2021	1302	Kermanshah	15.2 ± 1.85	‐	20.4	32.2
Naghavi^14^	2021	776	Kerman	22.2 ± 3.1	‐	33.4	‐
Ghasemipour^15^	2021	340	Rasht	‐	‐	‐	41
Alizadeh^16^	2021	322	Kerman	‐	‐	‐	17.4
Bahramnejad^17^	2020	609	Kerman	‐	‐	19.3	45.6
Bahramnejad^18^	2020	2676	Kerman	‐	‐	18.7	43.5
Shekari^19^	2020	3649	Tabriz	22.8	‐	9.1	‐
Karimi‐Afshar^20^	2020	384	Kerman	22.1 ± 2.5	‐	16.4	43.8
Karimirad^21^	2020	524	Hormozgan	23 ± 4.2	‐	11.1	‐
Kaveh^22^	2020	462	Shiraz	21.5 ± 3.1	‐	23.3	‐
Masjedi^23^	2020	1075	Varamin	14 ± 0.8	‐	‐	25.5
Pashapour^24^	2020	2261	Tabriz	15.5 ± 0.5	‐	3.5	32.1
Roustaeizade shooroki^25^	2020	400	Shahrebabak	‐	30	‐	‐
Nasirzadeh^26^	2020	823	Qom	15.2 ± 3.2	‐	14.9	‐
Narimani^27^	2020	215	Ardabil	21.2 ± 1.4	‐	47.9	‐
Salehi^28^	2020	380	Karaj	22 ± 2.6	‐	44.7	‐
Hamzehi^29^	2019	499	Qom	21.3 ± 4	‐	‐	9.9
Zebhi^30^	2019	185	Guilan	22.1 ± 1.3	‐	‐	30.2
Miri‐Moghaddam^31^	2019	500	Zahedan	21.2 ± 2.4	‐	31.2	‐
Mohammadpoorasl^32^	2019	4820	Tabriz	15.7 ± 0.7	‐	3.1	34.3
Kabir^33^	2019	4940	National	20.6 ± 2.4	‐	13.7	17
Rajabalipour^34^	2019	1196	National	15.9 ± 0.8	‐	18.9	35.5
Ansari^35^	2019	1094	Zahedan	‐	‐	8.2	36.1
Ghahremani^36^	2019	483	Shiraz	‐	‐	20.2	35.2
Pourramazani^37^	2019	600	Kerman	16.6 ± 1.1	‐	19.9	41.8
Ghaderi^38^	2019	169	Bojnord	21.1 ± 2.03	18.9	‐	‐
Pirdehghan^39^	2019	771	Hamedan	17.36 ± 1.9	‐	‐	24.2
Marin^40^	2019	1311	Tabriz	19.5 ± 2.7	‐	5.9	‐
Abbasi‐Ghahramanloo^41^	2018	524	Hormozgan	23 ± 4.2	14.5	‐	‐
Khani Jeihooni^42^	2018	157	Fasa	23.1 ± 2.5	‐	‐	32.3
Rahimi^43^	2018	432	Birjand	16.5 ± 0.8	‐	17.4	‐
Marzban^44^	2018	800	Qom	15.74	‐	61.29	‐
Ziaei^45^	2018	1517	Tabriz	‐	‐	11.9	21.6
Ataeiasl^46^	2018	1133	Tabriz	15.5 ± 0.5	‐	5.0	33.5
Anbarlouei^47^	2018	1282	Tabriz	15.5 ± 0.50	‐	3.1	35.1
Bashirian^48^	2018	730	Hamadan	16.4 ± 0.8	‐	26.3	36.4
Shahraki‐Sanavi^49^	2018	457	Zahedan	‐	‐	10.13	‐
Gorjian^50^	2018	302	Abadan	‐	‐	‐	25.5
Nakhostin‐Roohi^51^	2017	1878	Ardabil	24.0 ± 5.6	28.9	‐	‐
Nabipour^52^	2017	682	Kerman	21.4 ± 2.5	‐	24.9	48.3
Latifi^53^	2017	1012	Tehran	21.4 ± 2.7	‐	26.3	34.1
Tarrahi^54^	2017	1131	Khorramabad	19.6 ± 2.2	‐	2.3	21.7
Refahi^55^	2017	1014	Zahedan	‐	27.1	15.3	40.4
Rezaei^56^	2017	630	Jahrom	15.7 ± 0.9	‐	9.3	‐
Sahebihagh^57^	2017	521	Qazvin	19.6 ± 2.4	‐	4.0	31.5
Mohammadi^58^	2017	1837	Sanadaj	15.1 ± 0.8	‐	‐	36.2
Khayyati^59^	2017	4422	Tabriz	15.8 ± 1.1	‐	‐	15.4
Afrashteh^60^	2017	977	Bushehr	21.1 ± 2.37	16.1	‐	‐
Makvandi^61^	2017	400	Asad Abad	22.7 ± 3.3	‐	‐	32
Mozafarinia^62^	2017	422	Tehran	22.4	‐	‐	14.9
Safiri^63^	2016	1730	Tabriz	‐	‐	11.6	‐
Aghajani^64^	2016	400	Kashan	17.3 ± 0.4	‐	‐	21.9
Maghsoudi^65^	2016	390	Larestan	22.3 ± 2.4	22.66	‐	‐
Zahedi^66^	2016	1730	Kerman	‐	44.6	‐	‐
Joveini^67^	2016	306	Sabzevar	22.4 ± 2.5	‐	46.9	‐
Kabir^68^	2016	1959	Karaj	22.4 ± 4.5	‐	3.4	41.85
Bashirian^69^	2016	601	Kermanshah	16.4 ± 0.8	‐	17.1	36.1
Alami^70^	2016	200	Gonabad	21.6 ± 2.3	‐	‐	59.6
Abbasi‐Ghahramanloo^71^	2016	1992	Tehran	26.2 ± 3.1	17.8	8.9	26.6
Mohammadkhani^72^	2016	201	Najaf Abad	‐	‐	23.9	39.8
Pirdehghan^73^	2016	704	Yazd	17.6 ± 0.6	‐	31.1	41.3
Rahimzadeh^74^	2016	288	Kurdistan	‐	‐	‐	11.8
Khademalhosseini^75^	2015	1020	Shiraz	16.3 ± 4.3	‐	4.3	‐
Roohafza^76^	2015	5408	Isfahan	14.4 ± 1.7	‐	11.5	33.1
Hedayati‐Moghaddam^77^	2015	590	Mashhad	20.8 ± 1.5	‐	‐	10.5
Yaghubi^78^	2015	7330	National	‐	17.9	11.6	28.7
Babaei Heydarabadi^79^	2015	604	Tehran	23	‐	‐	42.9
Pishdad^80^	2015	175	Qazvin	22 ± 3.5	‐	‐	34.29
Allahverdipour^81^	2015	1837	Tabriz	22.1	‐	8.5	‐
Fakhari^82^	2015	5192	Tabriz	15.7 ± 0.7	18.5	6.0	44.9
Bagheri Nesami^83^	2015	200	Sari	21.6 ± 1.5	‐	‐	26.5
Karimy^84^	2015	170	Ahvaz	21.25 ± 2.9	‐	‐	33.5
Mohammad‐Alizadeh‐Charandabi^85^	2015	1524	Sanandaj	15.1 ± 1.0	‐	‐	10.4
Meysamie^86^	2015	2877	Tehran	16.21	‐	25.7	41.6
Rashid^87^	2015	1030	Tehran	16.14	‐	‐	51.5
Heydari^88^	2015	1149	Jahrom	21.1 ± 2.5	‐	9.2	24.02
Chaman^89^	2015	450	Shahroud	16.5 ± 1.1	‐	‐	33.1
Mohammadi^90^	2014	450	Babolsar	15.3 ± 0.5	‐	‐	51.7
Mohammadpoorasl^91^	2014	1837	Tabriz	22.1 ± 2.3	‐	8.5	39.2
Taremian^92^	2014	3582	Tehran	‐	‐	‐	25.7
Ebrahimipour^93^	2014	400	Mashhad	22.4	30.8	‐	88.75
Abedini^94^	2014	211	Bandar Abbas	15.4	‐	‐	18
Monirpoor^95^	2014	1053	National	22.5	‐	32.9	41.3
Esmaielzadeh^96^	2014	510	Qazvin	‐	‐	30.58	59.2
Bakhshani^97^	2014	1000	Zahedan	‐	21.5	‐	35
Miri^98^	2014	2371	Khorasan	17 ± 0.9	‐	‐	33.23
Askarian^99^	2013	600	Shiraz	21.5	‐	‐	25.8
Karimy^100^	2013	380	Zarandieh	16.7 ± 1.3	‐	17.3	28.9
Goreishi^101^	2013	1200	Zanjan	21.3 ± 2.3	‐	9	18.8
Rezakhani Moghaddam^102^	2013	977	Tehran	22.7 ± 3.6	‐	‐	27.75
Rezakhani Moghaddam^103^	2013	720	Tehran	22	‐	‐	23.3
Roohafza^104^	2013	812	Isfahan	21.6 ± 1.2	‐	19.5	‐
Zivari‐Rahman^105^	2012	520	Kerman	‐	‐	13.2	
Ghanbari Hashem Abadi^106^	2012	1565	Mashhad	‐	19.8	6.4	30.5
Dehdari^107^	2012	162	Tehran	22.3 ± 2.9	29	22.1	‐
Nazemi^108^	2012	1800	Shahroud	28.5 ± 3.5	‐	‐	6.77
Ghavidel^109^	2012	400	Nazarabad	17.3	‐	‐	46.7
Mardani^110^	2012	310	Bandar Abbas	21.3	‐	‐	24.8
Sharifirad^111^	2012	578	National	23.3 ± 2.3	‐	‐	67.2
Tavousi^112^	2012	433	Tehran	16.8 ± 0.7	‐	‐	62.1
Hajian^113^	2011	882	Babol	21.9 ± 1.4	‐	9.8	‐
Alaee Kharaem^114^	2011	447	Karaj	16.5 ± 1.3	‐	‐	53
Sabahy^115^	2011	1024	Kerman	20.6 ± 2.3	23.82	18.6	42.5
Nakhostin‐Roohi^116^	2011	181	Ardabil	‐	13.3	‐	‐
Ziaaddini^117^	2011	610	Kerman	17.9 ± 0.5	‐	‐	51.5
Zareipour^118^	2011	200	Tehran	23.7 ± 2.8	‐	‐	63.5
Ghafouri^119^	2011	296	Tehran	22 ± 3.0	‐	51	
Heydari^120^	2010	1271	Tehran	‐	‐	‐	48
Fayaz‐Bakhsh^121^	2010	958	Tehran	21.8 ± 2.3	25.1	16.5	30.3
Ramezankhani^122^	2010	4523	Tehran	14.7 ± 2.1	‐	‐	54.9
Dehghani^123^	2010	534	Yazd	22 ± 3.4	‐	15.9	
Taraghi Jah^124^	2010	4483	National	21.5 ± 4.7	‐	‐	40.3
Sahraian^125^	2010	971	Shiraz	22.3	6.3	3.6	
Nakhostin‐Roohi^126^	2010	2324	Ardabil	‐	‐	‐	35.6
Shokouhi^127^	2009	958	National	‐	‐	‐	30.2
Valipour^128^	2009	100	Broujerd	23	‐	‐	36
Barikani^129^	2008	700	Tehran	14.8 ± 1.4		‐	30.6
Taremian^130^	2008	2997	Tehran	‐	22.1	13.2	33.9
Sohrabi^131^	2008	8375	National	‐	20.7	13	30
Ziaaddini^132^	2008	860	Kerman	‐	‐	9.4	11.8
Momenan^133^	2007	4361	Tehran	15 ± 1.8	‐	25.7	56.9
Abedini^134^	2007	200	Bandar Abbas	22.4 ± 2.3	‐	‐	32.5
Momen‐nasab^135^	2007	700	Khoramabad	21.3 ± 2.8	‐	‐	29.7
Hashemi Mohammadabad^136^	2001	206	Yasuj	25 ± 2.2	‐	‐	4.7

**FIGURE 2:**
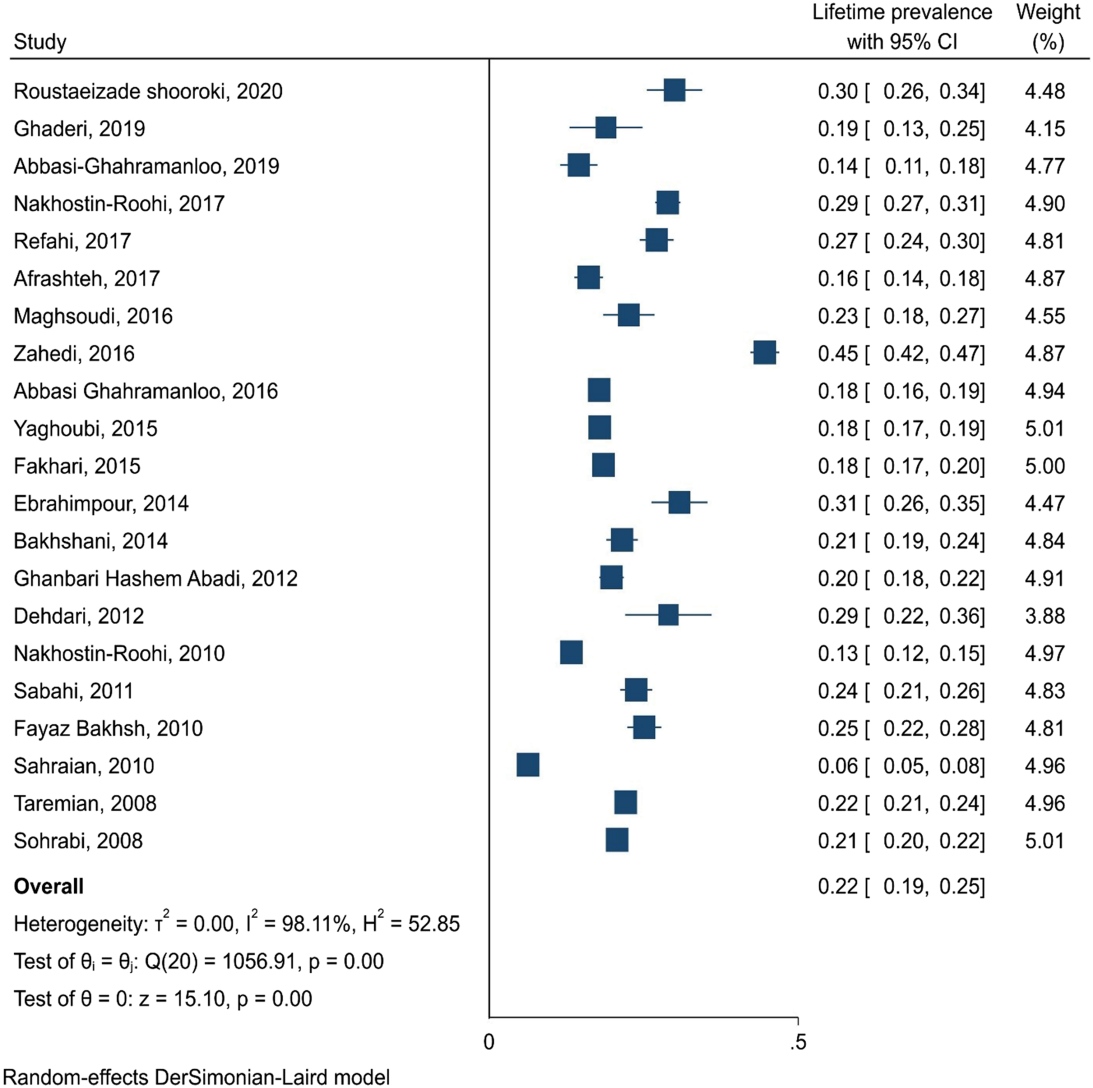
Period prevalence of hookah smoking in Iranian pupils and university students

The reviewed studies classified hookah smoking into three main categories, including lifetime prevalence (*n* = 92), point prevalence (*n* = 48) and period prevalence (*n* = 21). Correspondingly, the analysis of the findings indicated that the lifetime, period and point prevalence of hookah were 34.4% (95% CI: 31.5–37.2), 22% (95% CI: 19–25) and 18% (95% CI: 16–21), respectively (Figure 2). Based on the country's districts and type of the samples (high school and university students), the findings indicated that the lifetime prevalence of hookah smoking was higher in the high school students (34.4%) compared with the university students (32.3%). On the other hand, the point (21.5% vs. 16.3%) and period (22.5% vs. 20.8%) prevalence of hookah smoking was higher in the university students compared with the high school students. Based on the country's regions, the findings demonstrated Region 5 have the highest lifetime (41.7%; 95% CI: 33.5–50) and period prevalence (27.1%; 95% CI: 20.3–33.9) of hookah smoking. On the other hand, Region 1 had the highest point prevalence of hookah smoking (27.2%; 95% CI: 17.3–37.2). Table 2 shows the findings in more detail.

**TABLE 2:** The prevalence of hookah smoking by region and type of students

Subgroup			Number of studies	Prevalence (95% CI)	Between studies			Between subgroups	
					*I* ^2^	*P*_heterogeneity_	*Q*	*Q*	*P*_heterogeneity_
Lifetime prevalence	Region	1	29	37.5 (31.1–43.9)	99.49	0.001	5495.15	14.20	0.014
		2	13	26.3 (20–32.7)	98.02	0.001	605.69		
		3	15	29.3 (23–35.6)	99.36	0.001	2196.77		
		4	11	30.3 (26.9–33.7)	88.78	0.001	89.10		
		5	16	41.7 (33.5–50)	99.20	0.001	1877.46		
		National	8	36.2 (29–43.3)	99.44	0.001	1249.20		
	Type of students	Academic	51	32.3 (28.6–36)	99.19	0.001	6016.16	2.51	0.113
		High school	41	34.4 (31.5–37.3)	99.29	0.001	5664.67		
Period prevalence	Region	1	4	22.7 (19.8–26.4)	89.87	0.001	29.63	8.41	0.78
		2	4	14.7 (7.9–21.6)	96.68	0.001	900.33		
		3	3	20.2 (12.9–27.4)	98.69	0.001	153.20		
		5	8	27.1 (20.3–33.9)	97.69	0.001	30.31		
		National	2	19.3 (16.6–22)	94.94	0.001	19.77		
	Type of students	Academic	17	22.5 (19–26)	98.43	0.001	1019.17	0.39	0.532
		High school	4	20.8 (16.5–25)	91.71	0.001	36.17		
Point prevalence	Region	1	9	27.2 (17.3–37.2)	99.74	0.001	1517.99	32.43	0.001
		2	9	16.9 (12.2–21.6)	97.82	0.001	366.77		
		3	10	9.3 (6.8–11.8)	98.16	0.001	490.46		
		4	4	16.5 (3.9–29.1)	99.32	0.001	438.41		
		5	14	20.6 (16.4–24.7)	96.97	0.001	429.61		
		National	2	16.2 (11.1–21.3)	94.43	0.001	17.95		
	Type of students	Academic	22	21.5 (18–25.1)	98.74	0.001	1668.44	4.38	0.036
		High school	26	16.3 (12.9–19.7)	99.24	0.001	3177.53		

*Note*: Region 1: The provinces of Tehran, Alborz, Qazvin, Mazandaran, Semnan, Golestan and Qom; Region 2: The provinces of Isfahan, Fars, Boushehr, Chaharmahal va Bakhtiari, Hormozgan and Kohkilouyeh va Boyerahamad; Region 3: The provinces of Eastern Azarbaijan, Western Azarbaijan, Ardebil, Zanjan, Gilan and Kurdistan; Region 4: The provinces of Kermanshah, Ilam, Hamedan, Markazi and Khouzestan; Region 5: The provinces of Khorasan Razavi, Southern Khorasan, Northern Khorasan, Kerman, Yazd and Sistan va Balouchestan.

**FIGURE 3:**
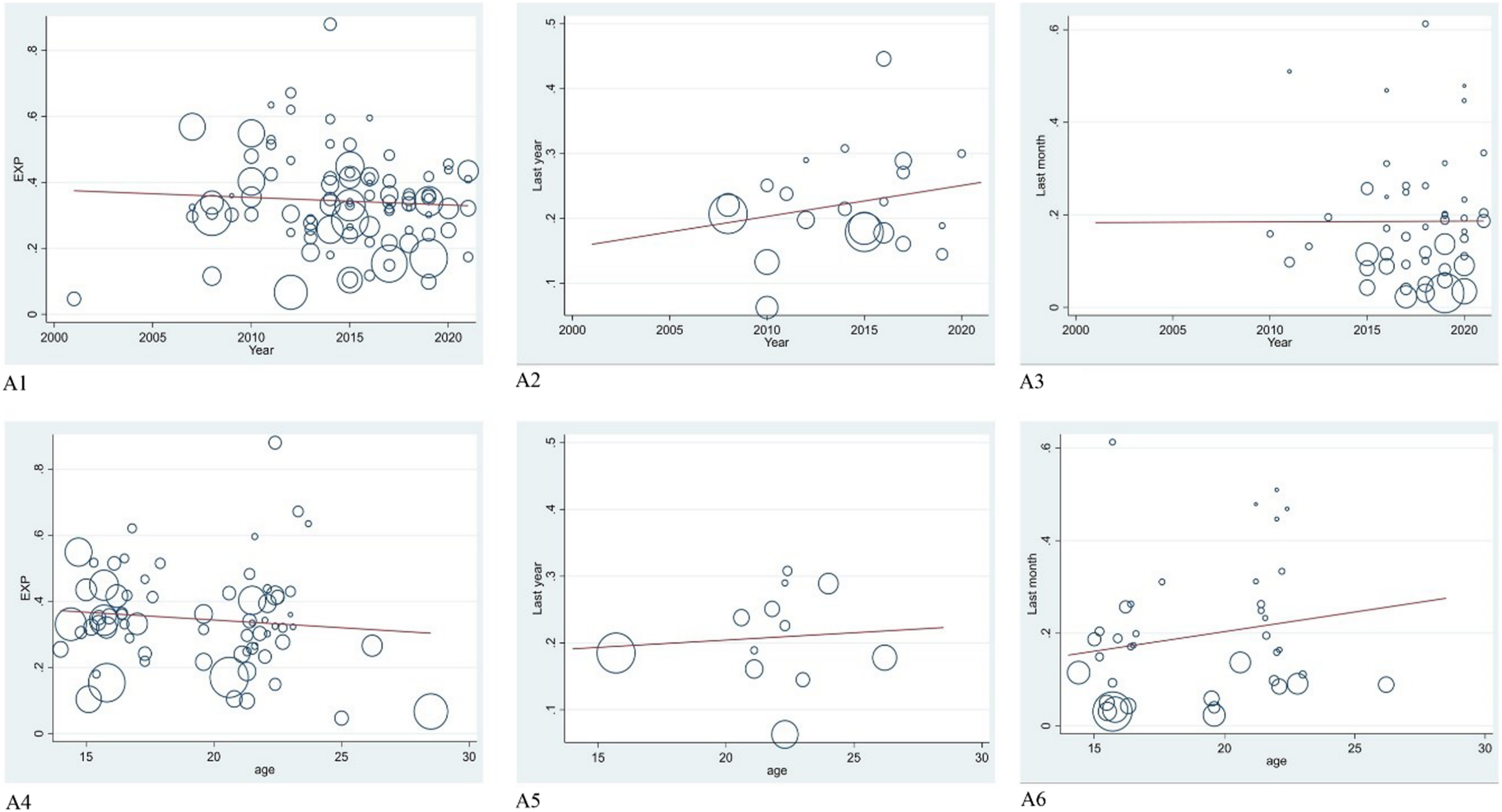
The results of meta‐regression. The relationship between year of publication with lifetime prevalence (A1), period prevalence (A2) and point prevalence (A3) and relationship between mean age of participants with lifetime prevalence (A4), period prevalence (A5) and point prevalence (A6). The size of the circles indicates the sample size of the articles

According to the results of meta‐regression analysis, the lifetime prevalence and period prevalence of hookah smoking were not correlated with the mean age of the participants, sample size and publication year. However, a significant correlation was observed between the point prevalence of hookah smoking and sample size (*P* = 0.015) (Figure 3).

**FIGURE 4:**
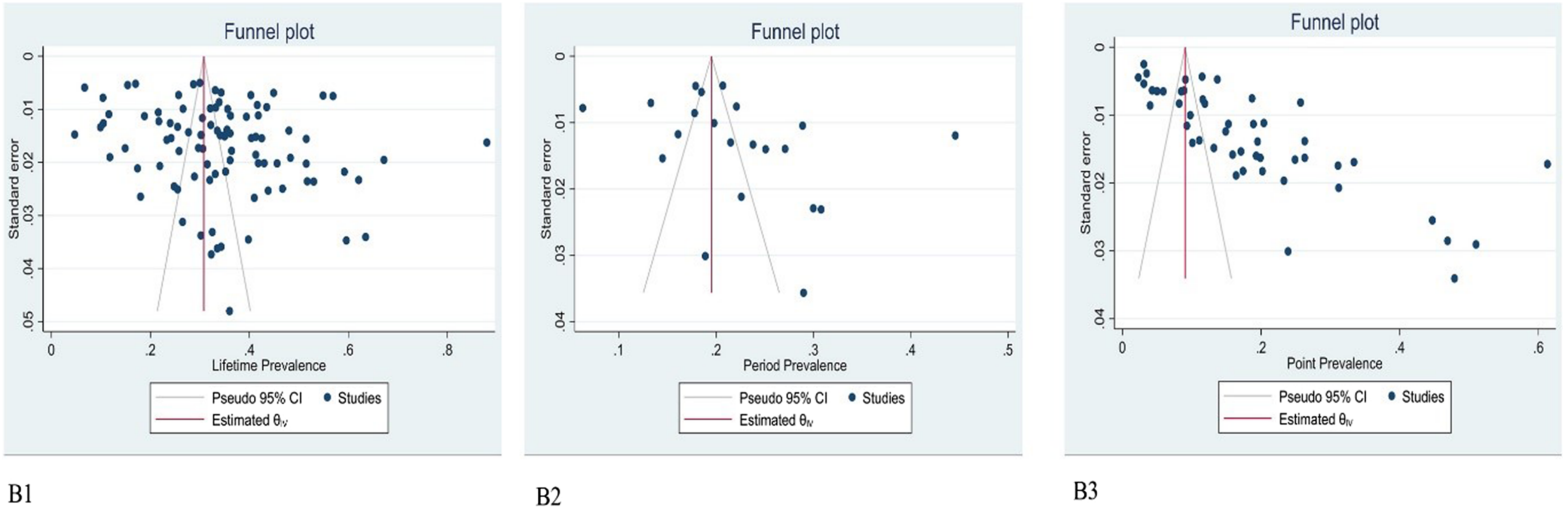
Publication bias for lifetime (B1), period (B2) and point (B3) prevalence of hookah smoking

One of the most important variables in meta‐regression analysis is the year of publication of the articles, because it shows how the prevalence of hookah use has changed over time. The lack of correlation between the year of publication and the prevalence of hookah indicates that all health advice and workshops held for students at universities and schools do not lead to a reduction in hookah use. One of the reasons for not reducing the prevalence of hookah is the misconception that the water in a hookah bottle is able to absorb nicotine and harmful substances, so hookah is not harmful. It seems that in order to reduce hookah use, it is necessary to correct these misconceptions and impose heavy penalties for hookah use in parks, promenades and even dormitories. Publication bias was not considered significant for the period prevalence of hookah smoking (*P* = 0.195), while it was significant for lifetime prevalence (*P* = 0.033) and point prevalence (*P* = 0.01) of hookah smoking (Figure 4).

## 4 DISCUSSION

The current meta‐analysis aimed to evaluate the prevalence of hookah smoking (lifetime, period and point prevalence) among Iranian pupils and university students. According to the findings, the lifetime prevalence of hookah smoking among Iranian pupils and university students was 34.4% and 32.3%, respectively. In this meta‐analysis, we used a precise and inclusive strategy to retrieve and analyse the largest number of studies possible without a time limit. Meanwhile, previous meta‐analyses in this regard evaluated fewer studies due to the lack of division based on lifetime, period and point prevalence.^137^, ^138^ In the previous two meta‐analyses, 58 and 37 studies were analysed, respectively, while a much larger number of studies were conducted in this field. However, due to the incomplete search, many studies were missed in the previous two meta‐analyses. Given that the decisions of health professionals are influenced by the findings of these studies, and poor findings can lead to wrong decisions, the implementation of this study (by eliminating the weakness of previous studies) seemed necessary. The number of studies that have examined the prevalence of hookah use in Iran is very large, which has a cultural aspect. In Iran, hookah is considered a recreational device that is used by families even in parks and recreation areas and even in some student dormitories, and despite health warnings, its use is still increasing. Consistent with our findings, the results of the meta‐analysis conducted by Aydogan et al indicated that the prevalence of hookah smoking was 31% among Turkish students.^139^ A similar study was performed on more than 2000 students in London, and the obtained results showed the lifetime prevalence of hookah smoking to be 40.1%, which is slightly higher than the estimated rate in the present study.^10^ In a cross‐sectional study conducted by Al‐Delaimy and Al‐Ani on 847 male high school students in Iraq, the prevalence of hookah smoking within the past 30 days was reported to be 46.1%, which is high than the current research. This discrepancy could be due to the fact that the mentioned study was performed on male students aged 15–18 years.^140^

According to a meta‐analysis, the prevalence of hookah smoking in university students in Saudi Arabia was 17%, which is a lower rate compared with the results of the present study.^141^ The high prevalence of hookah smoking in Iranian pupils and students could be attributed to cultural factors and general attitudes. Hookah is commonly served in Iranian ceremonies, and hookah is frequently carried around by Iranian families' outdoors as a recreational activity. In the current research, no significant correlation was observed between the prevalence of hookah smoking and the mean age of the participants. It seems that hookah smoking has become inherent to the youth culture. One of the main reasons is the misconception that hookah is less harmful than cigarettes because people believe that its toxins are dissolved by the water in the tank. In the present study, the results of meta‐regression analysis indicated no significant correlation between the prevalence of hookah smoking and the publication year of the studies. In other words, no ascending or descending trends were observed in this regard. Therefore, it could be concluded that the current measures taken to reduce the prevalence of hookah smoking are practically ineffective. The lifetime and point prevalence had a significant publication bias. The reason for this finding can be attributed to the tendency of journals to publish articles with specific results or that articles with specific results may not have been published.

One of the limitations of the present study was that data were not reported completely in the reviewed studies, which restricted our analysis. If the prevalence of hookah smoking had been reported based on gender and the lifetime prevalence, more practical findings would have been available. Also, in many articles, only one prevalence of hookah smoking was investigated and reported. Therefore, it is recommended that these limitations be addressed in further investigations in this regard. Despite these limitations, we analysed more data and a larger number of studies purposively, and the obtained results regarding the prevalence of hookah smoking in pupils and university students are more reliable.

## 5 CONCLUSION

According to the results, hookah smoking is highly prevalent among Iranian pupils and university students, who are the future of our country, and this unhealthy habit affects almost one third of these populations. Therefore, proper training should be developed in the form of educational workshops or through academic and school curricula in order to raise the awareness of youngsters regarding the adverse health effects of hookah smoking.

## CONFLICT OF INTEREST

The authors declare that they have no competing interests.

## ETHICS STATEMENT

Not applicable.

## AUTHOR CONTRIBUTIONS

HZ and RGG: concept development (provided idea for the research). YR and HS: search strategy. HZ, NK and RGG: data collection. RGG: supervision. YM: analysis/interpretation. All authors: writing (responsible for writing a substantive part of the manuscript).

## Data Availability

The datasets used and/or analysed during the current study are available from the corresponding author on reasonable request.
